# Prevalence and Traits of Mobile Colistin Resistance Gene Harbouring Isolates from Different Ecosystems in Africa

**DOI:** 10.1155/2021/6630379

**Published:** 2021-01-22

**Authors:** Madubuike Umunna Anyanwu, Charles Odilichukwu R. Okpala, Kennedy Foinkfu Chah, Vincent Shodeinde Shoyinka

**Affiliations:** ^1^Department of Veterinary Pathology and Microbiology, University of Nigeria, Nsukka 400001, Nigeria; ^2^Department of Functional Food Products Development, Faculty of Biotechnology and Food Science, Wroclaw University of Environmental and Life Sciences, Wroclaw, Poland

## Abstract

The mobile colistin resistance (*mcr*) gene threatens the efficacy of colistin (COL), a last-line antibiotic used in treating deadly infections. For more than six decades, COL is used in livestock around the globe, including Africa. The use of critically important antimicrobial agents, like COL, is largely unregulated in Africa, and many other factors militate against effective antimicrobial stewardship in the continent. Currently, ten *mcr* genes (*mcr*-1 to *mcr*-10) have been described. In Africa, *mcr*-1, *mcr*-2, *mcr*-3, *mcr*-5, *mcr*-8, and *mcr*-9 have been detected in isolates from humans, animals, foods of animal origin, and the environment. These genes are harboured by *Escherichia coli*, *Klebsiella*, *Salmonella*, *Citrobacter*, *Enterobacter*, *Pseudomonas*, *Aeromonas*, *Alcaligenes*, and *Acinetobacter baumannii* isolates. Different conjugative and nonconjugative plasmids form the backbone for *mcr* in these isolates; however, *mcr*-1 and *mcr*-3 have also been integrated into the chromosome of some African strains. Insertion sequences (ISs) (especially IS*Apl1*), either located upstream or downstream of *mcr*, class 1 integrons, and transposons, are drivers of *mcr* in Africa. Genes coding multi/extensive drug resistance and virulence are colocated with *mcr* on plasmids in African strains. Transmission of *mcr* to/among African strains is nonclonal. Contact with *mcr*-habouring reservoirs, the consumption of contaminated foods of animal/plant origin or fluid, animal-/plant-based food trade and travel serve as exportation, importation, and transmission routes of *mcr* gene-containing bacteria in Africa. Herein, the current status of plasmid-mediated COL resistance in humans, food-producing animals, foods of animal origin, and environment in Africa is discussed.

## 1. Introduction

Colistin (COL) is one of the few last-line antibiotics used in treating deadly infections caused by multidrug-resistant (MDR) and extensively drug-resistant (XDR) Gram-negative bacilli (GNB). The unrestricted use of antibiotics in human medicine, especially in form of self-medication that is predominant in low and middle-income countries, is a major cause of development of antimicrobial drug resistance [1]. The use of COL in humans was largely abandoned in the 1970s due to its neurotoxic and nephrotoxic effects alongside to the discovery and approval of new and effective antibiotics [2], but COL has been used in livestock for more than six decades in most countries in the world, including Africa [3–5]. In GNB, including *Enterobacterales* and nonfermenting GNB (such as *Pseudomonas*, *Acinetobacter*, and *Aeromonas*), the major mechanism of COL resistance is by the addition of 4-amino-4-deoxy-L-arabinose (L-Ara4N) and phosphoethanolamine (pEtN) to the lipid A, a moiety of lipopolysaccharide (LPS), which decreases the electrostatic interaction between COL and LPS [6, 7]. Before 2015, mutation in chromosomal genes, such as *pmrAB*, *phoPQ*, *mgrB*, and *ccrB*, were the known mechanisms of COL resistance. Uncommonly used in humans due to the availability of effective antibiotics, and considering the chromosomal genes would only transfer vertically to the progenies of a specific bacterial clones, there was a low interest in pursuing the COL resistance concept [8]. However, in the early 2000s, clinicians were forced to use COL in treating deadly infections caused by rapidly evolving and spreading MDR and XDR organisms [5, 9, 10]. Unfortunately, in late 2015, it was discovered that the clinical usefulness of COL is threatened by a plasmid-borne transmissible COL resistance determinant, mobile colistin resistance (*mcr*-1) [11]. Plasmids are highly mobile circular/linear extrachromosomal segments of DNA that can acquire insertion sequences, integrons, and transposons to disseminate resistance genes by horizontal gene transfer (HGT) [12].

There are ten *mcr* genes (*mcr*-1 to *mcr*-10) with many variants that have been detected in isolates from humans, animals, and environment across over 60 countries in 6 of the 7 continents except Antarctica [3, 5]. These genes encode MCR, which are transmembrane phosphoethanolamine (pEtN) transferases (proteins) conferring resistance to COL by adding a pEtN moiety to the lipid A of LPS in the outer membrane of GNB [13].

One Health paradigm involves surveillance of AMR resistance in humans, animals, and the environment [14–16]. Potentially pathogenic and resistant organisms emanating from either of these ecosystems move with ease to another [17]. The release of antimicrobials, antimicrobial-resistant bacteria, and ARGs by humans and animals (by ejection or by anthropogenic activities) into the environment results in the contamination of soil, aquatic systems, plants, and wildlife has serious ramifications on public health [17, 18]. Crops/plants (particularly vegetables and fruits) contaminated with COL-resistant organisms when consumed raw or undercooked by humans/animals pose a serious risk to the consumer's health [19]. Contaminated surface waters are considerable reservoirs for the transmission of COL-resistant organisms to the food chain in Africa because plants are irrigated and foods are processed with these waters.

COL resistance genes emerging from Africa, as any other continent, can be disseminated through international food (animal- and plant-based) trade and travel [20]. Thus, understanding the epidemiology, phenotypic, and genotypic characteristics of *mcr*-carrying isolates, the genetic context of *mcr* in the isolates, and population structure and mechanism of acquisition of *mcr* genes by organisms in Africa creates the needed impetus to tackle the problem to reduce the risk to public health [19]. Therefore, this current review articulates the findings of studies on plasmid-mediated COL resistance among isolates from different food-producing animals, foods of animal origin, humans, and environment in Africa.

## 2. Plasmid-Mediated Colistin Resistance among Isolates from Food-Producing Animals, Foods of Animal Origin, Environment, and Humans in Africa

### 2.1. Food-Producing Animals

COL has been used in the livestock sector in Africa for many decades. Thus, bacteria colonizing domesticated animals in Africa have developed resistance to COL. Twenty publications investigated plasmid-mediated COL resistance in 1096 faecal (rectal/cloacal) isolates from food-producing animals in Africa [21–40]. The studies reported *mcr*-1 gene in 199 isolates (197 *E*. *coli* and one each for *Citrobacter freundii* and *Alcaligenes faecalis*), *mcr*-5 gene in one *E*. *coli*, and *mcr*-8 gene in one each for *E*. *coli* and *K*. *pneumoniae*. Two of the studies probed *mcr*-1 directly in faecal samples [22, 33].

#### 2.1.1. Poultry

The poultry sector in Africa has been reported as a reservoir for plasmid-mediated COL resistance [22–24, 26–29, 31–34, 37–39]. In Northern Africa, *mcr*-1 was detected in 106/110 (96.4%), 16/109 (14.7%), 9/113 (8%), and 3/12 (25%) *E*. *coli* isolates from chickens/samples of chicken meat in Tunisia [24, 32, 34, 37, 39], Algeria [22, 26, 28, 33], Egypt [29, 31], and Morocco [38], respectively. This indicates that *mcr*-1 is widely circulating more among *E*. *coli* strains in the Tunisian poultry sector than in the other countries. It means that COL/other antimicrobial agents might be used more frequently in Tunisian livestock sector than in other Northern African countries. The *mcr*-1-positive isolates from the Egyptian poultry sector were isolated during 2010-2016, and they also contained virulence-associated genes (VAGs) and 18 additional resistance genes (including extended spectrum *β*-lactamase (ESBL) and plasmidic Ampicillinase C (pAmpC) genes) belonging to six antimicrobial families [29, 31] (Table 1), suggesting that organisms producing MCR-1 and ESBL/pAmpC have been present in Africa since at least 2010. Isolates from the Tunisian poultry sector carried *mcr*-1 on diverse plasmids (IncHI2, IncI2, 242 kb IncP, and IncFIB) [32, 34, 39], and they also possessed class 1 integrons and 21 antibiotic resistance genes (including ESBL and pAmpC genes) belonging to four antimicrobial families [24, 32, 34, 37, 39] while transposon IS*Apl1* was upstream of *mcr*-1 which was carried on IncHI2 plasmid in isolates from the Algerian poultry sector [28]. These findings suggest that diverse mobile genetic elements (MGEs) (plasmids, integrons, and transposons) facilitate the acquisition and transfer of *mcr*-1 in North Africa. It also suggested that the poultry sector in the region is a reservoir of cocktails of multiresistance genes, and that the localization of other resistance genes on *mcr*-1-plasmid favors the selection of COL-resistant strains under the selective pressure imposed by other antibiotics.

Some of the *mcr*-1-positive isolates from the Tunisian poultry sector were from poultry farms that imported chickens from France or derived their flocks from French chicks [24], suggesting that food animal trade is a route for the importation of plasmid-mediated COL resistance into Africa. However, the animals could also have been contaminated after their arrival or subsequent rearing stages in Tunisia [20]. The *mcr*-1-positive isolates from the Tunisian poultry sector were extensively diverse, belonging to phylogroups A, B1, B2, and D and 16 STs [32, 34, 39] (Table 1), including zoonotic high-risk (HiR) extraintestinal pathogenic *E*. *coli* (HiR-ExPEC) clones ST69, ST10, and ST117 [41], while strains from the Algerian poultry sector were of ST48 and ST758 [28, 33], suggesting that commensal and virulent *mcr*-1-harbouring *E*. *coli* clones are circulating in the livestock sector in North Africa. The ST48 *E*. *coli* is a progenitor of various clones considered human associated [42], highlighting the importance of monitoring COL resistance from a One Health perspective to understand the exchange of *mcr* gene-containing bacteria (MGCB) between human-animal-environmental ecosystems in Africa.

Furthermore, *mcr*-1 was directly detected in faecal samples of 28 of 623 chickens (0.6%) in Algeria [22, 33], suggesting that livestock/animal manure is a potential source for dissemination of COL-resistant organisms to humans (especially those that handle animals/make contact with animal manure) and the environment. Eleven *mcr*-1-carrying strains were detected among 104 *E*. *coli* (10.6%) recovered from the 28 *mcr*-1-positive samples [22, 33], suggesting that direct testing of samples for *mcr* followed by isolation is the best approach for surveillance of plasmid-mediated COL resistance in an ecological niche.

In South Africa, 38 *mcr*-1-carrying strains were detected among 191 *E*. *coli* (19.9%) isolated in 2015 from chickens [23, 27], suggesting that *mcr*-1 is widely spread among *E*. *coli* in Southern Africa since at least half a decade ago. IS*Apl1* was upstream of *mcr*-1 which was located on IncI plasmid in some strains, and there were six other resistance genes in three antimicrobial families [27] (Table 1), suggesting that IncI is a common driver of COL resistance in South Africa. It is likely that *mcr* originated in the livestock sector in South Africa since the use of COL in human medicine has been restricted as a schedule 4 drug (obtained only by prescription) in the country, whereas its use in livestock especially poultry was not restricted until 2016 [43].

In Nigeria, *mcr*-1.1, *mcr*-5.1, and *mcr*-8 were detected in 10 strains (nine *E*. *coli* and one *Citrobacter freundii*), one *E*. *coli*, and eight *K*. *pneumoniae*, respectively, among 34 isolates (55.9%) from liver/cloacae of poultry birds [40], suggesting that diverse *Enterobacteriaceae* are circulating cocktails of *mcr* genes in West Africa, which causes infections with limited choices of antibiotic therapy. The *mcr*-1.1 was located on IncX4 plasmid, and there were more than one *mcr* gene types (*mcr*-1 and *mcr*-8) as well as extra resistance genes (including ESBL, pAmpC, and plasmid-mediated quinolone resistance (PMQR)) belonging to four antimicrobial families in the *mcr*-positive strains (Table 1), suggesting that IncX4 is a common driver of COL resistance in Nigeria, and that organisms coproducing MCR, ESBL, and pAmpC are present in the Nigerian livestock industry. Unfortunately, conjugation was positive, implying that *mcr*-1.1 is transferable to other organisms. It is also documented that a novel *mcr*-1 variant, named *mcr*-1.22, was detected in an *E*. *coli* isolated from chicken in Nigeria (Genbank accession no. MN017134), thus further indicating that the livestock sector in West Africa is a reservoir for *mcr* genes. The presence of diverse *mcr* genes in the Nigerian poultry sector is due to the fact that poultry farmers in the country frequently use cocktails of antimicrobials, most of which contain COL for prophylactic control/treatment of intestinal infections [44]. Thus, a policy on the restriction of nontherapeutic COL use is urgently warranted in Nigeria. However, a chromosomal mutation in *pmrB* was detected in *K*. *pneumoniae* strains isolated from poultry in Nigeria [22], suggesting that COL resistance in West Africa is also mediated by the chromosomal mechanisms. It is also worth noting that *mcr*-1.1 was detected in three*E*. *coli* recovered from chickens in Tanzania (https://www.ncbi.nlm.nih.gov/pathogens/antimicrobial-resistance/), suggesting that *mcr*-1 is circulating in the livestock sector in East Africa. Nevertheless, it is worthy to note that none of the three isolates recovered in 2010-2012 from chickens in South Africa harboured *mcr*-1 [43]. Furthermore, none of the 93 ESBL-producing and COL-resistant *E*. *coli* isolated in 2011 from chickens in Senegal contained *mcr*-1 or *mcr*-2 [45]. However, mutation in chromosomal *pmrA* and *pmrB* genes was observed in some of the strains, further suggesting that chromosomal genes are also involved in COL resistance in West Africa.

#### 2.1.2. Pigs

African porcine sector has been reported as a potential reservoir for MGCB [30, 40]. In South Africa, a porcine ST446/phylogroup A ESBL-producing *E*. *coli* isolate carrying *mcr*-1 (on diverse plasmids) as well as 12 other resistance genes (including ESBL and PMQR genes) (Table 1) was isolated from a pig at a slaughterhouse [30]. The organism also harboured VAGs, including *ast* encoding heat-stable enterotoxin 1 associated with human diarrhoea [46], suggesting that the South African pig industry is a potential reservoir for virulent multi- to extensively drug-resistant organisms. The presence of diarrhoeagenic *mcr*-habouring *E*. *coli* in the African food chain is worrisome. This is because the treatment of food-borne diarrhoea, which is one of the most common causes of mortality among rural Africans, could be difficult if associated with MGCB. In Nigeria, one *mcr*-1-carrying *Alcaligenes faecalis* was detected among 12 faecal multidrug-resistant (MDR) isolates (8.3%) recovered in 2016 from the rectum of pigs [40], suggesting that non-*Enterobacteriaceae* has been circulating *mcr*-1 in West Africa since at least 2016. COL selective pressure in the Nigerian porcine sector is likely due to the use of various antimicrobials (including colistin) without the veterinarian's supervision in the management of pigs [47]. Prophylactic antibiotic use against meningitis and sepsis (which are relatively common economic diseases in pigs caused by diverse organisms) is a potential cause of antimicrobial resistance in pigs [48]. However, other possible sources of COL-resistant organisms in the African porcine sector include slaughterhouse waste (blood meal and internal organs) and fly larvae (maggots) grown in animal faeces that are often used as an economical mode for feeding of pigs [49].

#### 2.1.3. Cattle

The presence of MGCB in the beef/dairy cattle sector in Africa has been reported [21, 25, 35, 50]. *mcr*-1 gene was detected in eight COL-resistant *E*. *coli* (isolated from cattle/other unreported sources) in South Africa [21]. ESBL (*bla*_CTX-M-1_) and pAmpC (*bla*_CMY-2_) genes were present in some of the strains, suggesting that organisms producing MCR-1, ESBL, and pAmpC have been present in Southern Africa. Studies from Northern Africa also reported plasmid-mediated COL resistance in the beef/dairy sector. In Egypt, an MDR ExPEC ST10 strain carrying *mcr*-1 on a nonconjugative plasmid and four other resistance genes belonging to two antimicrobial families (Table 1) was detected among 38 *E*. *coli* (2.6%) isolates from cows with subclinical mastitis [25], suggesting low prevalence of *mcr*-1 among clinical *E*. *coli* strains from Egyptian diary sector, and that *mcr*-1 is vertically transferred among highly successful zoonotic HiR pandemic clones circulating in Egypt. However, the *mcr*-1-positive strain was susceptible to cephalosporins, carbapenems, and aztreonam, highlighting the need for antimicrobial stewardship in Africa to retain the efficacy of common and critically important antibiotics.

In Tunisia, 14 faecal strains carrying *mcr*-1 on IncP, IncHI, and IncFIB plasmids and class 1 integrons were detected among 25 ESBL-producing *E*. *coli* (56%) isolated from cattle [35, 50], suggesting that there is a high prevalence of *mcr*-1-habouring *E*. *coli* in the Tunisian bovine sector, and that diverse MGEs evolved *mcr*-1 and ESBL genes in commensal and virulent *E*. *coli* clones in North Africa. Four other resistance genes (including ESBL gene) in three antimicrobial families were also present in the *mcr*-1-positive strains [35, 50] (Table 1), suggesting that virulent multiresistant *E*. *coli* strains are colonizing food-producing animals in the Mediterranean region. Interestingly, some of the *mcr*-1-carrying strains were recovered from calves kept in a farm where COL was used to treat diarrhoea [35], suggesting that diverse plasmid easily evolves from COL selective pressure. The *mcr*-1-positive strains were extensively diverse belonging to six STs (dominated by ST162) [35] (Table 1), including pandemic HiR-ExPEC zoonotic clones ST10 and ST88 [41], suggesting that commensal and virulent *E*. *coli* clones producing MCR-1 and ESBL are acquired from contaminated animal environment (such as skin of dams during suckling, feed, herbage, and drinking water).

In Nigeria, one *mcr*-1-carrying *E*. *coli* was detected among seven COL-resistant *E*. *coli* (14.3%) isolated in 2016 from rectal swabs of cattle [40], further suggesting that *mcr*-1 has been present in the bovine sector in West Africa since at least four years ago. The use of cocktails of antimicrobials (most of which contain COL) in treating undiagnosed diseases by nomadic cattle herders and nonprofessionals without veterinarian's supervision [44] is the possible cause of COL selective pressure in the Nigerian bovine sector. However, the animals might have acquired MGCB through the consumption of contaminated water/herbage since cattle are reared extensively in Nigeria.

#### 2.1.4. Camels

Camels have been reported as reservoirs of COL-resistant organisms in North Africa [36]. In a Tunisian study, one MDR ST162/phylogroup B1 ESBL-producing strain carrying *mcr*-1 and ESBL genes on a 260 kb IncHI2 plasmid was detected among 179 faecal *Enterobacteriaceae* (0.6%) from butchery camels [36], suggesting that there is low circulation of *mcr*-1 among *Enterobacteriaceae* colonizing camels in Tunisia and that IncHI2 is a driver of *mcr*-1 and ESBL gene in the dromedary sector in Tunis. The ST162 *mcr*-1-positive *E*. *coli* has also been isolated from chickens and calves in Tunisia [34, 35], suggesting that ST162 is a widely spread *mcr*-1-harbouring clone in Tunisia. The presence of MGCB in the dromedary population in Africa poses a serious danger to public health, especially to the camel handlers/tourists who make contact with them and handlers/consumers of meat from these camels. Nevertheless, it is worthy to note that none of the 52 faecal *Enterobacteriaceae* isolated in 2011-2013 from camel (*Camelus dromedarius*) calves in Tunisia carried *mcr*-1 or *mcr*-2 genes [51]. Similarly, none of 162 *E*. *coli* isolated in 2017 from dromedary camels in Kenya harboured *mcr*-1 to *mcr*-5 [52]. In Nigeria, none of the three and two COL-resistant *E*. *coli* isolated from camels and dogs, respectively, contained *mcr*-1 to *mcr*-8 [40].

### 2.2. Foods of Animal Origin

Handling and consumption of contaminated foods of animal origin is a potential route for dissemination of antimicrobial resistance to human and animal populations. Inadequate husbandry and slaughterhouse facilities and unhygienic slaughtering techniques facilitate the contamination of animal-related products in Africa [53]. Six publications investigated the *mcr* gene in 211 isolates from foods of animal origin in Africa [36, 50, 54–57]. Twenty-nine strains were reported to harbour *mcr*-1 among the isolates tested.

#### 2.2.1. Meat

Studies from North Africa reported the presence of COL-resistant organisms in samples of raw meats/ready-to-eat (RTE) meat products [34, 56]. In Tunisia, 15 strains carrying *mcr*-1 on IncP, IncFIB, and IncI1 plasmids and class 1 integrons were detected among 30 ESBL-producing and COL-resistant *E*. *coli* (50%) isolated from meat samples collected from retail stores/supermarkets [34], suggesting that diverse MGEs facilitate the spread of plasmid-mediated COL resistance through the food chain in Tunis, and that *mcr*-1 is widely spread in the poultry meat production/supply chain in North Africa. The *mcr*-1-positive strains were extensively diverse, belonging to four STs (dominated by ST57) (Table 2), including the pandemic HiR-ExPEC zoonotic clone ST117 [41], and they also contained 10 additional resistance genes (including ESBL and heavy metal genes) belonging to five antimicrobial families (Table 2), suggesting that handling and consumption of contaminated meat is a potential source for the acquisition of commensal and virulent clones habouring genes coding against last-resort antimicrobial(s) in Africa.

In Egypt, nine *E*. *coli* carrying *mcr*-1 and one carbapenemase-producing (*bla*_VIM-1_) *Enterobacter hormaechei* (isolated during 2017) carrying *mcr*-9 were detected among 129 COL-resistant *Enterobacteriaceae* (0.8%) isolated from samples of raw meat/RTE meat products collected from supermarkets, slaughterhouses, and butcher's shops [55–57], suggesting that farm-to-fork transmission of MGCB in Africa is facilitated by cross-contamination of meat in slaughterhouses (critical point of contamination by potentially multi to extensive resistant organisms). The *mcr*-1 and *mcr*-9 were on 64.4 kb IncI2 and 250 kb IncHI plasmids, respectively, and there were 22 other resistance genes (including ESBL, PMQR, and heavy metal resistance genes) belonging to five antimicrobial families in the *mcr*-positive strains [55–57] (Table 2). This suggests that uncooked and half raw foods of animal origin are a source for the dissemination of multiresistant organisms coproducing MCR, ESBL, and carbapenemase in Africa, and that IncHI2 is a major driver of *mcr*-9/heavy metal resistance along the food production and supply chain in the Middle East. The *mcr*-1-positive strains were extensively diversified belonging to eight STs [56, 57] (Table 2), including pandemic and zoonotic HiR-ExPEC clones ST10 and ST58 (which dominated) [41], and conjugation was positive for *mcr*-1 and both *mcr*-9 and carbapenem-resistance encoding gene (*bla*_VIM-1_) [56, 57], implying that farm-to-plate transmission is a route for the dissemination of virulent *mcr*-containing strains capable of transferring genes encoding resistance against last-resort antimicrobials to other organisms (potentially acquired through handling/consumption of uncooked food of animal origin) in Africa. The presence of organisms resistant to COL and carbapenems in the food chains is alarming because diseases associated with these strains are often hard-to-treat and often are untreatable. In Africa, carbapenems are not used in livestock but are used in human medicine [58]; therefore, carbapenem resistance in the food chain possibly emanated from human setting through contamination of food animals and associated products. This warrants improvement in the hygiene of slaughterhouses and their personnel in Africa. Nevertheless, it is worth mentioning that none of the 36 COL-resistant *Enterobacteriaceae* isolated from raw beef and chicken meat sampled in Egypt harboured *mcr*-1 [59].

#### 2.2.2. Dairy Products (Milk and Cheese)

North African studies documented the presence of COL-resistant organisms in raw and ready-to-eat dairy products [50, 54]. In Tunisia, one ExPEC ST10 carrying *mcr*-1 on IncP and IncFIB plasmids was detected among five ESBL-producing *E*. *coli* (20%) isolated from samples of raw bovine/caprine milk collected from bulk-holding tanks/containers [50], suggesting that a considerable percentage of isolates from milk produced in Tunis is *mcr*-1 carriers. It also suggested that IncP and IncFIB plasmids are the common drivers of *mcr*-1 in the Mediterranean region, and that uncooked foods of animal origin (milk) are potential sources for the acquisition of MGCB. The presence of *mcr*-1-positive ST10 *E*. *coli* in food of animal origin as well as its presence in livestock [25, 34, 39], human [60], and the environment [61] in North Africa suggests that it is a highly disseminated COL-resistant ExPEC clone in the region. There were seven other resistance genes (including ESBL and heavy metal resistance genes) belonging to five antimicrobial families in the *mcr*-1-positive strain (Table 2), further suggesting that the food chain is a route for the dissemination of virulent disinfectant-resistant COL-resistant organisms in Africa. The presence of disinfectant-resistant organisms in the food chain portends a substantial danger to public health, especially to handlers and consumers of raw dairy products. The use of disinfectants for washing drinking troughs and milk collection/holding containers and as foot dips in livestock farms are putative sources for heavy metal selective pressure in bacterial organisms [62]. Contaminated udder, teat, and hands of milkers are also potential sources of heavy metal-resistant organisms in milk [63].

In Egypt, *mcr*-1 flanked by IS*Apl1* and 14 other resistance determinants (including a heavy metal gene) were detected on IncHI2 plasmid in one pandemic HiR-ExPEC zoonotic clone ST69 among four COL-resistant *E*. *coli* (25%) isolated from RTE raw milk cheese [49]. The organism also harboured *astA* genes, meaning that milk is also an important reservoir for highly virulent multi-/disinfectant-resistant organisms capable of causing difficult-to-treat diseases (diarrhoea) in Africa. It also suggested that *mcr*-harbouring organisms are likely evading biosecurity measures (such as disinfectant foot dips) in farms in Africa. This highlights the need for assessment of the quality of disinfectants used in livestock farms in Africa. However, possible sources of antimicrobial-resistant organisms in cheese include from contaminated animal skin (udder/teat), hands of milkers, flies, or fomites that enter into the milk during the milking process as well as from containers and preparers of the cheese [63].

### 2.3. Environment (Manure/Soil, Aquatic, and Wildlife Ecosystems)

The environment is the receptacle of anthropogenic and agricultural wastes laden with antimicrobial-resistant organisms. It is also established that the environment constitutes a substantial source for the evolution of antimicrobial-resistant organisms [64]. Thus, other ecosystems interface with the environment from/to where resistant organisms can be easily acquired or deposited. Nine publications reported on plasmid-mediated COL resistance in 582 isolates from the environment in Africa [40, 61, 65–71]. These studies reported *mcr*-1 gene-type variants in 66 isolates (51 *E*. *coli*, four *K*. *pneumoniae*, seven *Pseudomonas aeruginosa*, one *Pseudomonas fluorescens*, and three *Pseudomonas* species), *mcr*-2 in five isolates (one *P*. *aeruginosa* and two each for *E*. *coli* and *K*. *pneumoniae*), and *mcr*-3 in two *E*. *coli* strains.

#### 2.3.1. Animal Manure/Soil Ecosystem

Dissemination of plasmid-mediated COL resistance into the manure/soil ecosystems has been documented in North Africa [61]. In an Algerian study, three ST10 *mcr*-1-carrying and two ST155 *mcr*-3-carrying strains were detected among 22 MDR COL-resistant *E*. *coli* isolated from agricultural soil [61]. In the same study, two ST405 strains were detected among 20 MDR COL-resistant *E*. *coli* (10%) isolates from animal (horse and cattle) manure. These findings suggest that pandemic HiR-ExPEC zoonotic clones have disseminated diverse *mcr* genes from the livestock ecosystem through animal manure into the soil ecosystem in Algeria. There was an ESBL gene in some of the strains [61] (Table 3), suggesting that organisms coproducing MCR-1 and ESBL have disseminated into various environmental ecological niches in North Africa. It also suggested that pandemic HiR-ExPEC zoonotic clones [41] producing ESBL and MCR-1 might be circulating in the Algerian equine and bovine industries. However, since COL is not known to be used in horses in Tunisia, COL selective pressure in Tunisian equine sector possibly emanated from the use of other antibiotics [72]. Nevertheless, the animals could also have acquired organisms from persons (such as groomers, caretakers, and jockeys) who are often in contact with them and/or through the consumption of contaminated herbage or fluid. It is very likely that MGCB in the sampled agricultural soil in Algeria originated from untreated or insufficiently treated animal manure used as organic fertilizer in the farmlands. Runoffs can potentially carry MGCB from the soil to aquatic ecosystems (such as surface waters and aquacultures), and rainfall drops could also raise resistant organisms from the soil to plants, thereby contaminating these niches and posing a risk to public health [19]. Encouragingly, it has been reported that composting and anaerobic digestion is effective in eliminating *mcr* in animal manure [73]. However, garbage flies could also have picked the COL-resistant organisms from other places and subsequently deposited them on the animal manure during feeding/breeding [19]. However, more worrisome is that conjugation was positive, implying that the isolates could rapidly transfer *mcr*-1 to other organisms. Therefore, individuals (humans and animals) who make contact with animal manure and soil as well as those who handle/consume raw/undercooked plant products (such as vegetables) are at great risk of acquiring *mcr* genes. Unfortunately, in Africa, the majority of farmers work manually without personal protective equipment and vegetables are often consumed raw or undercooked. Thus, farmers and consumers of raw/undercooked contaminated vegetables/farm crops in Africa are at greater risk of infection by COL-resistant organisms present in manure/soil.

#### 2.3.2. Aquatic Ecosystem

*(1) Wastewater*. Wastewaters are contaminated fluids from anthropogenic and agricultural settings, which contain nutrients that support the growth of bacteria and rapid exchange of MGEs [74]. Thus, wastewaters are established sources of new emerging pathogenic antimicrobial-resistant organisms. Wastewaters have been reported as a reservoir of MGCB in Africa [65, 66, 71]. Thirty-one MDR *E*. *coli* carrying *mcr*-1 and *daaE* gene (associated with human diarrhoea) were isolated from municipal wastewater effluents in South Africa [65], suggesting that COL-resistant potentially escapes the wastewater treatment methods used in South Africa. This finding of *mcr*-positive organisms in wastewater is worrisome because the health of the public is in danger as this insufficiently treated wastewater may eventually be released into surface water bodies and farmlands thereby contaminating terrestrial/aquatic wildlife, soil, and humans through contact with the environment or food chain. Thus, improvement in wastewater treatment protocols in Africa is warranted.

In the Republic of Congo, eight *mcr*-1-carrying strains (four *P*. *aeruginosa*, one *P*. *fluorescens*, and three *Pseudomonas* spp.) were detected among 20 MDR *Pseudomonas* (20%) isolated in 2017 from community wastewaters [66], suggesting that *mcr*-1 has disseminated in the human population in East Africa since at least three years ago. Because of the poor wastewater treatment facilities in Congo-Brazzaville (like in most sub-Saharan African countries), MGCB emanating from human wastewaters can easily disseminate into other ecosystems. Thus, there is a need for improved hygiene and waste management in Congo-Brazzaville, a country whose infrastructural facilities were overstretched due to a prolonged/recurrent civil war.

In Tunisia, one ST8900/phylogroup A *E*. *coli* carrying *mcr*-1 and an ESBL gene (*bla*_CTX-M-15_) on IncFIB and IncP-1 was detected among seven ESBL-producing and COL-resistant *Enterobacterales* (14.3%) isolated from raw wastewater collected from a wastewater treatment plant (WWTP) receiving wastewater from diverse sources (domestic, hospital, urban, rain, and industrial waters) [71]. This suggests that diverse promiscuous plasmids have widely spread *mcr*-1 among *Enterobacterales* colonizing humans in Tunisia, and that environmental dissemination of plasmid-mediated COL resistance in the country originates from anthropogenic/agricultural settings. IS*Apl1* and *pap2* flanked the *mcr*-1 upstream and downstream, respectively, while class 1 integrons harbouring other resistance genes, including heavy metal resistance gene, were also present in the *mcr*-1-positive strain (Table 3). This implies that diverse genetic elements facilitate the spread of *mcr*-1 in North Africa, and that WWTPs are hotspot collectors of MDR *Enterobacterales* in the Mediterranean region. This further highlights the need for adequate treatment of wastewaters before discharge into the environment.

*(2) River and Seawaters*. The presence of MGCB in natural water in North Africa has been reported [68]. In Algeria, two tigecycline-resistant *E*. *coli* of ST115 and ST23 strains carrying *mcr*-1.1 on IncHI2A and *mcr*-1.5 on IncI2 plasmid, respectively (Table 3), were detected among 246 COL-resistant isolates (0.8%) from seawater polluted with domestic, hospital, agricultural, and industrial wastes [68, 75], suggesting that pandemic HiR-ExPEC zoonotic clones [41] have disseminated from anthropogenic/agricultural settings (due to improper disposal of wastes) into water bodies in the Mediterranean region. The worse is that the strains contained 14 resistance genes in four antimicrobial families [75] (Table 3), indicating that seawater in Africa is a reservoir of cocktails of multiresistance genes. Surface waters containing COL-resistant pose a substantial threat to public health, especially to the coastal dwellers in Africa that use these waters for various purposes including food processing, recreation, drinking, fishing, laundering, and bathing [19]. Remarkably, the organisms did not transfer *mcr*-1 to a recipient organism [68], suggesting that the plasmidal location of a *mcr* gene does not necessarily imply its transferability. Since tigecycline is the last-line antibiotics used for treating deadly infections, the presence of tigecycline-resistant and COL-resistant organisms in the natural environment can complicate the dynamics of antimicrobial resistance in Africa. Thus, surveillance of tigecycline resistance in diverse ecological niches in Africa is warranted. It is also worth noting that *mcr*-5.1 and novel *mcr*-3 genes, named *mcr*-3.33 to *mcr*-3.37, were detected in *Aeromonas* strains recovered from rivers (receiving wastes from communities) and storm waters in South Africa (https://www.ncbi.nlm.nih.gov/pathogens/antimicrobial-resistance/), suggesting that in South Africa, environmental waters are reservoirs of *mcr*-3 and *mcr*-5 possibly originating from anthropogenic/agricultural settings.

*(3) Irrigation Water*. The presence of COL-resistant bacteriain irrigation water has also been reported in North Africa. An MDR ST345 *mcr*-1-carrying strain was detected among 31 COL-resistant *E*. *coli* (3.2%) isolated from irrigation water in Algeria [61], further suggesting that *mcr*-1 disseminated from agricultural/anthropogenic settings into surface waters in Algeria. Contaminated irrigation water is a source for the contamination of plants and soil, thereby posing health risks to farmers and consumers of raw/undercooked plant materials. Moreover, MGCB in the Algerian irrigation system can disseminate into the Mediterranean Basin, contaminating farmlands and plants, thus posing a risk to public health [19].

#### 2.3.3. Wildlife

Wildlife has been reported as a potential reservoir for COL-resistant organisms in Africa. In Algeria, a faecal ST405/phylogroup D *mcr*-1-carrying *E*. *coli* was isolated from a Barbary macaque monkey (*Macaca sylvanus*) [67], suggesting that a pandemic HiR-ExPEC zoonotic clone is circulating COL resistance in the wild in North Africa. Genes encoding ESBL and PMQR were also present in the strain, suggesting that wild animals in Africa are potential disseminators of genes conferring resistance to last-resort antibiotics. The genome of the ST405 strain did not conjugate with that of a recipient organism, suggesting that the *mcr*-1 might be located on the chromosome or nonconjugative plasmids, hence can be vertically transferred and maintained/persist in the wild. Since antibiotics are not used in wildlife, anthropogenic and agricultural wastes that contaminated plants or fluids consumed by the monkey are possible sources of *mcr*-1 in the wild [19]. The ST405 *E*. *coli* clone is associated with global dissemination of ESBL [67]. MCR-1-producing ST405 ExPEC clone was associated with diseases in humans in Algeria [76, 77], and it has been found in livestock and the environment in the country [28, 61], thus suggesting that this organism is circulating in human/animal/environmental ecosystems in North Africa.

The role of wildlife in the dissemination of *mcr* in the environment and human population has been reported in Africa [69]. In Egypt, 15 (12.3%) *mcr*-1-carrying strains (nine *E*. *coli*, three each for *K*. *pneumoniae*, and *P*. *aeruginosa*) and three (2.5%) *mcr*-2-carrying strains (one each for *E*. *coli*, *K*. *pneumoniae*, and *P*. *aeruginosa*), respectively, were detected among 122 isolates from wild (resident/migratory) birds [69], suggesting that diverse organisms (*Enterobacteriaceae* and non-*Enterobacteriaceae*) habouring different *mcr* gene types are colonizing wildlife in North Africa. The resident birds probably picked the MGCB from an environment contaminated with anthropogenic/agricultural wastes, while the migratory birds possibly picked organisms from other places and transported them to Algeria. More worrisome is the fact that three (16.7%) *mcr*-1-carrying (two *E*. *coli* and one *K*. *pneumoniae*) and two (11.1%) *mcr*-2-harbouring (one each for *E*. *coli* and *K*. *pneumoniae*) strains were detected among 18 isolates from environmental waters, which were in close proximity to the habitations of the wild birds, suggesting that the birds possibly disseminated the COL-resistant organisms into these waters. Even worse is that two (6.5%) strains (one each for *E*. *coli* and *K*. *pneumoniae*) carrying *mcr*-1 and three (9.7%) strains (one *E*. *coli* and two *K*. *pneumoniae*) carrying *mcr*-2 were detected among 31 isolates (16.1%) from humans living close to the water bodies, suggesting that these individuals might have acquired *mcr* genes from the contaminated waters. Anthropogenic (laboratory, home, and human wastes from swimmers and ships) and agricultural runoffs are also potential sources of COL-resistant organisms in the surface waters. There were both *mcr*-1 and *mcr*-2 in some isolates from migratory wild birds and humans, suggesting that surface waters in Africa are potential reservoirs for cocktails of *mcr* genes, thus posing a risk to public health. It also suggested that in Africa, the aquatic environment is a good medium for rapid acquisition and transfer of *mcr* genes.

African wildlife has been noted as potential vectors for intercontinental dissemination of COL-resistant organisms. One ST48 strain carrying *mcr*-1 on a 65 kb IncI plasmid was detected among 29 faecal ESBL-producing *E*. *coli* (3.4%) isolated from fennec foxes (*Vulpes zerda*) exported to China from Sudan [70], suggesting that organisms coproducing MCR-1 and ESBL have disseminated into the wildlife in Sub-Saharan Africa, and that wildlife trade is a route for dissemination of plasmid-mediated COL resistance. More worrisome was the fact that the strain transferred *mcr*-1 to a recipient organism, and it also contained 11 resistance genes, including genes encoding resistance to *β*-lactams (*bla*_TEM_ and *bla*_CTX-M-64_), fosfomycin (*fosA*), tetracycline (*tetA*), folate-pathway antagonists (*sul1*, *sul2*, and *dfrA*), aminoglycosides (*mphA* and *mdf*), phenicols (*floR*), and PMQR (*oqxB*). This means that wildlife is a potential reservoir for organisms that can transfer genes encoding resistance to last-resort antimicrobials to other organisms, thus posing a risk to public health, especially to those that interact (handle/consume) with wildlife. It was not reported whether the foxes were fed and/or given water before sampling in China. *mcr*-1 is highly endemic in China, especially in foods of animal origin with which the foxes could have been fed. Nevertheless, *mcr*-1 is very likely originated from the agricultural/anthropogenic setting in Sudan. Thus, there is a need for surveillance of COL resistance during imported wildlife quarantine. It is worthy to mention that *mcr*-1 was not detected in one COL-resistant *K*. *pneumoniae* isolated from the hospital environment in Nigeria [40].

### 2.4. Humans

Humans interface with animal and environmental ecosystems. Thus, they can deposit (through various anthropogenic activities) and acquire (through consumption of and contact with contaminated foods, fomites, and environment) antimicrobial-resistant organisms from these other ecosystems. Thirty-two studies probed *mcr* in 1,320 isolates from humans in Africa and reported *mcr*-1 gene-type variants in 113 isolates (24 K. *pneumoniae*, 71 *E*. *coli*, 15 *Pseudomonas*, two *Acinetobacter baumannii*, and one *Enterobacter*), *mcr*-8 in 6 K. *pneumoniae*, and *mcr*-9 in two *Enterobacter hormaechei*.

The dissemination of MGCB in human populations in Sub-Saharan Africa has been reported [40, 66, 77–87]. Five *mcr*-1-carrying strains (three *P*. *aeruginosa* and one each for *P*. *luteola* and *P*. *putida*) were detected among 14 clinical MDR *Pseudomonas* (35.7%) isolated from persons in the Republic of Congo [66], suggesting that *mcr*-1 is widely spread among non-*Enterobacteriaceae* circulating in the human population in Central Africa. Overuse of antimicrobials due to increased traumatism and high disease burden resulting from a prolonged and persistent civil war are factors that possibly prompted the development of COL-resistant organisms in Congo-Brazzaville.

The presence of MGCB in hospitalized individuals in East Africa has been reported [78–81]. In Sudan, 13 strains (seven *E*. *coli*, five *K*. *pneumoniae*, and one *Pseudomonas*) carrying *mcr*-1 were detected among 285 clinical MDR *Enterobacteriaceae* (4.6%) isolated in 2016 from individuals [78, 79], suggesting low circulation of *mcr*-1 among diverse organisms (*Enterobacteriaceae* and non-*Enterobacteriaceae*) colonizing individuals in East Africa since at least four years ago. In Kenya, a hospital-associated HiR ST15 K. *pneumoniae* (isolated during 2017) which harboured *mcr*-8 and 27 other resistance genes (including ESBL, PMQR, and fosfomycin resistance genes) (Table 4) on IncHI1B and IncR plasmids was isolated from a patient [80], indicating that uncommon plasmids are spreading *mcr* among virulent organisms in Africa, and that hospitalization (due to lack of antimicrobial stewardship and poor healthcare infrastructures) is a potential risk for the acquisition of MGCB in the continent. In Tanzania, 11 ST46 strains carrying *mcr*-1.1 (on a 33 kb IncX4 plasmid) and 18 other resistance genes (including ESBL gene) (Table 4) were detected among 20 faecal COL-resistant MDR *E*. *coli* (55%) isolated in 2017 from healthy persons working in hotels in Zanzibar Tanzania [81]. This indicates high community transmission of organisms coproducing MCR-1 and ESBL in Tanzania since at least 2017, and that places frequented by tourists in Africa are fertile niches for the exchange of MGCB between the hosting community population and the travelers. Possible causes of gut colonization of hotel workers by COL-resistant organisms include frequent empirical use of drugs without prescription, uncontrolled use of antibiotics, including COL in food and animal settings, and limited sanitation of the food chain [81].

Evidence that *mcr* genes has disseminated into the human ecosystem in West Africa has been documented [40, 82]. Nine (five *K*. *pneumoniae* and four *E*. *coli*) and three strains carrying *mcr*-1 and *mcr*-8 (*K*. *pneumoniae*), respectively, were detected among 78 clinical *Enterobacteriaceae* (17.9%) from individuals in Nigeria [40, 82] indicating that diverse *mcr* genes have disseminated into the human ecosystem in West Africa. It is very likely that MGCB disseminated from the livestock sector to the human population in Nigeria since COL for human medicine is not known to be used in the country.

South African studies also reported the circulation of *mcr* in the human population [83–87]. Twenty-five strains (17 *E*. *coli* and five *Klebsiella* carrying *mcr*-1 and three ESBL-producing COL-susceptible *Enterobacter hormaechei* carrying *mcr*-3) were detected among 59 clinical *Enterobacteriaceae* (42.4%) isolated from individuals in South Africa [83–87], indicating high prevalence of *mcr* among diverse *Enterobacteriaceae* colonizing humans in South Africa. The *mcr*-1 was flanked upstream by IS*Apl1*/IS*28* on 30-75 kb IncI2, IncHI2, and IncX4 plasmids [84], *mcr*-9 was also on IncHI2 plasmid [87], and there were 40 antimicrobial determinants (including ESBL and pAmpC genes) in the *mcr*-positive strains. The *mcr*-1-positive *E*. *coli* isolates belonged to six STs (dominated by ST624) [83–85] (Table 4), including pandemic HiR-ExPEC zoonotic clone ST10 [41]. These findings suggest that diverse genetic elements (especially IncHI plasmids) drive *mcr* and genes coding resistance to last-resort antimicrobials in commensal and virulent clones in South Africa. Some of the *mcr*-1-positive strains transferred *mcr*-1 to a recipient organism [84], suggesting that *mcr*-1 could be rapidly transferred to other organisms. Interestingly, some of the individuals who yielded *mcr*-1-positive strains had never received COL therapy, and some of them had never received antibiotic treatment [83]. Thus, suggesting that the dissemination of organisms producing MCR-1 into the human population in South Africa is very likely originated from the livestock sector. However, prior visits to the hospital for more than six months, carbapenem and fluoroquinolone therapy, antiviral therapy, and organ transplant were factors that could also have prompted the acquisition of *mcr*-1 by the organisms [83]. It was also reported that some of the *mcr*-1-positive strains exhibited carbapenem resistance, but one of them was susceptible to all tested antimicrobials [86], thus suggesting that the *mcr* genes can be harboured by COL-resistant isolates that are susceptible to other antibiotics. Furthermore, the COL susceptibility exhibited by *mcr*-9-habouring *Enterobacter* strains is associated with the lack of *qseB*/*qseC* gene, which induces COL resistance in *mcr*-9-containing strains [88]. However, the strains possessed mutations in the chromosomal genes *mgrB* and *pmrB*, further suggesting that *mcr* and chromosomal genes are responsible for COL resistance in Africa. Although the *mcr*-9-positive strains were related, they differed from all other African isolates, suggesting that they have been imported into South Africa, possibly following medical tourism to other countries. Therefore, screening and surveillance of tourists, especially those who visited areas with high *mcr* endemicity, are warranted. The colonization of humans in Sub-Saharan Africa by MGCB calls for serious concern; this is because these organisms could easily disseminate into the environment/other ecosystems following unhygienic practices like open air defecation which is still common in the region.

Studies from North Africa also reported humans as reservoir for COL-resistant organisms [28, 60, 76, 77, 89–97]. In Egypt, 20 strains (six *E*. *coli*, nine *Pseudomonas aeruginosa*, three *K*. *pneumoniae*, and two *Acinetobacter baumannii*) carrying *mcr*-1 and one tigecyline-resistant *Enterobacter hormaechei* (isolated during 2015) carrying *mcr*-9 on IncHI2 plasmid were detected among 285 clinical MDR isolates (7%) from individuals [89–95], suggesting low circulation of *mcr* genes among *Enterobacteriaceae* and non-*Enterobacteriaceae* causing difficult-to-treat diseases in Egypt, and that organisms producing MCR-9 had been present in North Africa, since at least half a decade. It also suggested that organisms in the ESKAPE (*Enterococcus*, *Staphylococcus*, *K*. *pneumoniae*, *Acinetobacter baumannii*, *P*. *aeruginosa*, and *Enterobacter*) group, which are highest-priority clinically relevant organisms, are circulating *mcr* genes in Africa. The presence of *mcr*-carrying strains of ESKAPE (especially *P*. *aeruginosa* and *A*. *baumannii*) in Africa portends a substantial challenge to public health (due to increased disease burden and cost of hospital stay/treatment) because various intrinsic and acquired traits enable these organisms to exhibit resistance to many antibiotics, including the critically important ones, and they represent the most causes of difficult-to-treat nosocomial infections worldwide [98, 99]. Some of the *mcr*-1-positive strains were recovered from individuals without a travel history [91], persons with healthcare-associated infections [89], and those who took antibiotics one month before hospitalization [95]. Thus, suggesting that antibiotic consumption is a putative risk for the development of carbapenem-resistant and COL-resistant bacteria, and that these organisms are disseminated from the community to the hospitals and *vice versa* in Africa. Class 1 integron was present in one *mcr*-1-positive *E*. *coli* [91], and there were IS*903* and IS*1* upstream and downstream of *mcr*-9, respectively. Moreover, there were 15 extra resistance genes (including ESBL, carbapenemase, and PMQR genes) belonging to five antimicrobial families in the *mcr*-positive strains [91, 94] (Table 4). These findings indicate that diverse genetic elements (plasmids, transposons, and integrons) facilitate the acquisition and transfer of *mcr* and genes coding resistance to last-resort antimicrobials in Africa. Unfortunately, the genome of some of the *mcr*-1- and *mcr*-9-positive strains conjugated with that of recipient organisms [91, 94], implying that the genes could be transferred to other organisms (easily in the gut), thus posing a worrisome risks to public health. Since MCR-9/carbapenemase-producing *Enterobacter hormaechei* has also been detected in food of animal origin in Egypt [57] and humans in South Africa [87], it means that *Enterobacter* is a prolific trafficker of *mcr*-9 and that handling/consumption of contaminated animal-related products is a potential route for transmission of this organism into the human ecosystem in Africa.

In Algeria, four *E*. *coli* (isolated in 2011) carrying *mcr*-1 and one virulent ST3336 K. *pneumoniae* (isolated in 2018) which harboured *mcr*-8 and 15 other resistance determinants (including ESBL, carbapenemase, and PMQR genes) in six antimicrobial families (Table 4) were detected among 276 clinical *Enterobacteriaceae* (1.8%) isolated from individuals, including a person without history of travel [28, 76, 77, 96, 97], suggesting that there has been a low community transmission of organisms coproducing MCR, ESBL, and carbapenemase in human population in Algeria since at least nine years ago. This portends a grave danger to public health due to the possibility of limited antimicrobial therapy for managing infections associated with organisms coproducing MCR, ESBL, and carbapenemase. The *mcr*-1 was flanked downstream by IS*Apl1* on 140 kb IncFIB and IncP plasmids in some of the strains belonging to the ExPEC clone ST405 [28, 76, 77], thus suggesting that diverse genetic elements (transposons and plasmids) evolved *mcr*-1 among pandemic HiR-ExPEC zoonotic clones causing difficult-to-treat diseases in humans in Algeria. The *mcr*-1-positive ST405 *E*. *coli* is very likely disseminated into Algerian human and environmental ecosystems from the animal sector since COL is frequently used in livestock husbandry in Algeria [28]. Unfortunately, the ST405 *E*. *coli* strain can rapidly transfer *mcr*-1 to other organisms, having transferred the gene to a recipient organism at a frequency of 10^–7^ cells/recipient [28, 76, 77]. Nonetheless, one of the *mcr*-1-positive isolates did not transfer *mcr*-1 to a recipient organism, suggesting that the gene was located on the chromosome or nonconjugative plasmid in the *mcr*-1-positive strain; hence, it can be vertically transferred and maintained in the human population, thereby posing a worrisome risk to public health. Moreover, it was reported that *mcr*-1 was stable in the isolates after 30 subculture passages [28], suggesting that the gene can persist in different ecological niches for a long time.

The role of travelers in the importation of *mcr* into Africa has been reported [60]. *mcr*-1 was detected in rectal swab samples of 23 among 440 individuals (5.2%) who performed the pilgrimage (Hajj) to Mecca after their return to their home countries of Morocco and Algeria [60], suggesting that overly crowded multinational events represent a fertile niche for the rapid transmission of *mcr* between individuals who subsequently carry these genes back to their countries [20]. Only 10 *Enterobacteriaceae* (nine *E*. *coli* and one ST788 K. *pneumoniae*) carrying *mcr*-1 were isolated from the 23 *mcr*-1-positive samples, suggesting that by isolation alone, the magnitude of plasmid-mediated COL resistance in an ecological niche can be underestimated. Extensive diversity existed among the *mcr*-1-positive *E*. *coli* isolates, which belonged to six STs dominated by pandemic HiR-ExPEC zoonotic clones ST10 and ST648 (Table 4), suggesting that traveling is a route for dissemination of virulent *E*. *coli* clones capable of causing difficult-to-treat diseases.

African residents have been noted as potential transporters of *mcr* to other places [100]. An ESBL-producing *Enterobacter cloacae* harbouring *mcr*-1 was isolated from an Algerian hospitalized in France [100]. Although it was reported that the patient did not travel to Algeria for many years, there is a possibility that the patient's gut was colonized before traveling to France since there was no reported history of the patient having contact with livestock in France. Thus, there is a need for a globally coordinated effort to combat the spread of the *mcr* genes through travel [20].

Traveling to Africa has also been noted as a putative risk of acquisition of MGCB [101, 102]. An ESBL-producing pandemic HiR-ExPEC zoonotic clone ST410 carrying *mcr*-1 on IncI plasmid was isolated from the urine samples of an American who traveled to Kenya and China [101], further suggesting that virulent MGCB can be acquired in Africa through the consumption of foods of animal origin or contact with livestock/fomites contaminated with COL-resistant organisms. However, there is also a possibility that the American acquired the organism in China. An ESBL-producing *E*. *coli* and *Salmonella* Virchow carrying *mcr*-1 (flanked upstream by IS*Apl1*) on IncI2 and IncHI2 plasmids, respectively, were isolated in the United Kingdom from patients who traveled to Egypt [102], further supporting that travelers to Africa can contract MGCB from the region and transport them back to their countries.

## 3. Conclusions

**Figure 1:**
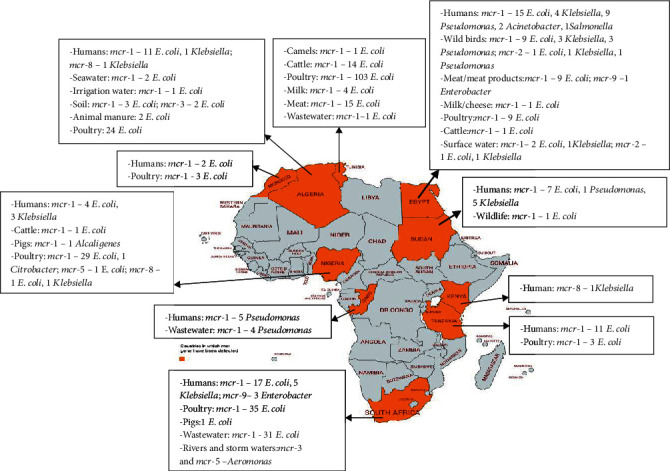
Distribution of organisms containing *mcr* genes in Africa. This map was created using an online service (https://mapchart.net/).

This review showed that the diversity of organisms such as *E*. *coli*, *Salmonella*, *Klebsiella*, *Citrobacter*, *Enterobacter*, *Pseudomonas*, *Aeromonas*, *Acinetobacter baumannii*, and *Alcaligenes faecalis* habouring various *mcr* genes is widely spread in humans, animals, food of animal origin, and environmental (manure/soil, aquatic, and wildlife) ecosystems in Africa (Figure 1). *E*. *coli* is the predominant organism spreading *mcr* genes in Africa, and *mcr*-1 is the most trafficked COL resistance gene in the continent. The oldest *mcr-*containing isolates from Africa are *E*. *coli* isolated in 2010 from chickens in Egypt [29]. In Asia, the oldest *mcr*-harbouring isolates were *E*. *coli* recovered in 1980 from food-producing animals in China [103], while the oldest *mcr*-containing isolates from Europe were *E*. *coli* strains of bovine origin recovered in 2004 in Italy [104]. Inappropriate use of antimicrobial agents in humans and animals, anthropogenic activities such as defecation in open environment/water and bathing/swimming in water bodies, and improper disposal of untreated as well as insufficiently treated animal manure, slaughterhouses, home, hospital, and laboratory wastes are the major causes of development of COL-resistant organisms and dissemination of *mcr* genes in Africa. Circulation of COL-resistant bacteria in human population in Africa is concerning because, if carbapenemase genes are present in the isolates, COL is one of the only options left for the treatment of human infections [105]. The presence of MGCB in natural water bodies in Africa is evidence that the anthropogenic burden of COL use resulted in environmental contamination in the continent. This can complicate the transmission dynamics of COL-resistant organisms and the rate of evolution of plasmid-mediated COL-resistant strains. Isolates from Africa carry *mcr* genes, together with many virulence and resistance genes, including those coding resistance to critically important antimicrobial agents. These organisms are superbugs that can potentially cause difficult-to-treat infections with pandemic potential. Some African isolates have acquired mega-plasmids with numerous ARGs (some harbour ≥ 10 genes). The presence of *floR* gene encoding florfenicol (an antibiotic that is only used in veterinary medicine) resistance in some African isolates (Table 4) suggests that veterinary usage of COL possibly prompted the acquisition of *mcr* in the isolates. Thus, a policy on the ban of nontherapeutic COL use is urgently warranted to curb the development and spread of plasmid-mediated COL resistance in Africa. Since there is very limited access to effective antimicrobials capable of destroying COL-resistant organisms in Africa, the development of effective antimicrobial stewardship programs and the use of nonantibiotic alternatives such as probiotics and antimicrobial peptides in the management of livestock are crucial to prevent an increase in the development of COL resistance and prevalence of MGCB in Africa. Since some of the *mcr*-negative isolates in this review may contain *mcr* genes that were undetectable by screening methods used in the various studies, surveillance monitoring of *mcr* using high-throughput techniques (such as whole genome sequencing) is important to determine the actual magnitude of PMCR in Africa.

Diverse genetic elements, including conjugative and nonconjugative plasmids, class 1 integrons, transposons, and insertion sequences, are driving the horizontal/lateral transmission of plasmid-mediated COL resistance in Africa. IncHI2, IncI2, IncFIB, and IncP are the predominant plasmids facilitating the spreading of *mcr* genes in Africa. The *mcr* genes carried by African strains can spread worldwide since the genes were transferred to recipient strains. Nonetheless, *mcr*-1 and *mcr*-3 have integrated into the chromosomal DNA and nonconjugative plasmids in African strains, enabling their vertical transmission and persistence among clonal lineages. Transmission of the *mcr* genes among strains from Africa is nonclonal even among diverse zoonotic virulent pandemic enterobacterial clones. More worrisome is that *mcr* has disseminated into the wild in Africa; this is an indication that *mcr* is maintained in bacterial populations in Africa regardless of antimicrobial selective pressure. Indeed, through horizontal/lateral and vertical transfer, *mcr* genes (*mcr*-1, *mcr*-2, *mcr*-3, *mcr*-5, *mcr*-8, and *mcr*-9) have spread widely into diverse ecosystems in Africa. These *mcr* genes are potentially exported from Africa to other continents through international travel, animal/plant, and food trade. This further underlines the need for globally coordinated One Health approaches.

## Figures and Tables

**Table 1:** Studies reporting plasmid-mediated colistin resistance in domesticated animals in Africa.

Source of isolate	Country	Year of isolation (*mcr* gene assayed)	Number of isolates tested for *mcr*	Identified gene/variant (number of organism)	Sequence type and/or phylogroup (virulence genes)	Plasmid (associated insertion sequence)	Additional resistance traits	References
Chickens	Tunisia	2013-2018 (*mcr*-1 to *mcr*-8)	141	*mcr*-1 (103 *E*. *coli*)	ST2197, ST10, ST69, ST349, ST57, ST1011, ST4187, ST3882, ST5693, ST8932, ST4187, and ST5693 (A1, B2, and D)	IncI1, IncI2, IncFIB and IncP, IncHI2 and IncP	*Int*1, *bla*_CTX-M-1_, *bla*_CMY-2_, *bla*_TEM_, *bla*_SHV_*bla*_CTX-M-55_, *bla*_CTX-M-14_, *bla*_CMY_, *bla*_TEM-1b,_*tet*(*A*), *tet*(*B*), *dfrA*, *cmlA*, *sul1*, *sul3*, *aadA*, *dfrIA*, *floR*, *strA*, *strB*, and *aadA1*	[26, 32–34, 37]
	Algeria	2015-2016 (*mcr*-1)	117	*mcr*-1 (24 *E*. *coli*)	ST48, ST5758, and ST758	IncHI2 (IS*Apl1*)	-	[22, 26, 28, 33]
	Egypt	2010-2016 (*mcr*-1 and *mcr*-2)	113	*mcr*-1 (9 *E*. *coli*)	A, F, and B1 (*cma*, *hemL*, *iroN*, *iss*, *pic*, *vat*, *hylE*, *ireA*, and *mchF*)	-	*bla*_TEM_, *bla*_CMY_, *bla*_CTXM_, *sul2*, *sul3*, *dfrA1*, *dfrA14*, *tet*(*A*), *cmlA1*, *aadA1*, *aphA*, *mphA*, *mrx*, *strB*, *floR*, *mphA*, and *aphA*	[29, 31]
	Nigeria	2019 (*mcr*-1 to *mcr*-8)	290	*mcr*-1 (7 *E*. *coli* and 1 *Citrobacter werkmanii*), *mcr*-5 (1 *E*. *coli*), and *mcr*-8 (1 *E*. *coli* and 1 K. *pneumoniae*)	ST2485 and others	IncX4	*tet*(*G*), *tet*(*D*), *tet*(*C*), *bla*_TEM-93_, *bla*_TEM-57_, *bla*_CTX-M-55_, *bla*_CMY-47_, *bla*_TEM-1_, and *qnrB17*	[40]
	Morocco	2012-2017 (*mcr*-1)	12	*mcr*-1 (3 *E*. *coli*)	-	-	-	[38]
	South Africa	2015 (*mcr*-1)	44	*mcr*-1 (38 *E*. *coli*)	-	IncI2 (IS*Apl1*)	*tet*(*A*), *sul3*, *dfrA12*, *aadA1*, *strA*, and *strB*	[23, 27]
Cattle	Tunisia	2016-2018 (*mcr*-1 to *mcr*-5)	25	*mcr*-1 (14 *E*. *coli*)	ST1642, ST10 ST162, ST88, ST533, and ST744 (B1)	IncP, IncFIB, and IncHI2	*Int*1, *bla*_CTX-M-1_, *bla*_TEM-1B_, *tet*(*A*), and *sul1*	[35, 50]
	Egypt	2014 (*mcr*-1)	38	*mcr*-1 (1 *E*. *coli*)	ST10	Nonconjugative plasmid	*bla*_TEM-1_, *dfrA-orF-aadA2*, and *aadA22*	[25]
	Nigeria	2016-2019 (mcr-1 to mcr-8)	65	*mcr*-1 (1 *Alcaligenes faecalis)*	-	IncX4	*-*	[40]
Camels	Tunisia	2016-2018 (*mcr*-1)	179	*mcr*-1 (1 *E*. *coli*)	ST162	IncHI2	*bla*_CTX-M-1_ and *bla*_CTX-M-15_	[36]
Pigs	South Africa	2016 (*mcr*-1)	1	*mcr*-1 (1 *E*. *coli*)	ST446 and A (*ast* and *gad*)	IncHI2, IncHI2A, and IncN	*bla*_TEM-1B_, *bla*_CTX-M-55_, *aacA4*, *aadA5*, *aac*(*6*′)*-Ib-cr*, *OqxB*, *fosA*, *tet*(*A*), *floR*, *drfA7*, *drfA17*, and *sul2*	[30]
	Nigeria	2016-2019	65	*mcr*-1 (1 *Alcaligenes faecalis*)	-	-	*-*	[40]

*mcr*: mobile colistin resistance gene; -: no data; additional resistance traits: resistance factors identified in one or pooled *mcr*-positive isolate; sequence type: Warwick multilocus sequence type of all *mcr*-positive *E*. *coli* isolates; plasmid: plasmid types in one or pooled *mcr*-containing isolates; Inc: incompatibility; IS: insertion sequence.

**Table 2:** Studies reporting plasmid-mediated resistance in foods of animal origin in Africa.

Source of isolate	Country	Year of isolation (*mcr* gene assayed)	Number of isolate tested for *mcr*	Identified gene/variant (number of organism)	Sequence type and/or phylogroup (virulence genes)	Plasmid (associated insertion sequence)	Additional resistance trait	References
Raw meat/ready-to-eat meat products	Egypt	2017-2019 (*mcr*-1 to *mcr*-8)	137	*mcr*-1 (9 *E*. *coli*) and *mcr*-9 (1 *Enterobacter hormaechei*)	ST101, ST58, ST1172, ST109, ST155, ST1720, ST1431 and ST10, and B1 *Enterobacter*: ST279 (*usp*, *OmpT*, *fimA*, *fimH*, *fimA*, *ETTT*, *EAST1*, *cdt*, *fyuA*, *traT*, *aer* and *iron*, *fimH86*, *cma*, *ireA*, *gad*, *lpfA*, and *iss*)	IncI2, IncHI2/IncHIA	*bla*_TEM-1_, *bla*_TEM-116_, *bla*_CTX-M-28_, *bla*_CTX-M-28,_*bla*_VIM-1,_*bla*_ACT-_16, *floR*, *dfrA12*, *aac*(*3*′)-*IIa*, *aph*(*3*)-*Ib*, *aadA2*, *aac*(*6*′)-*Ib-cr*, *aac*(*6*′)*-Il*, *∆aadA22*, *mphA*, *mdfA*, *tet*(*A*), *sul1*, *sul2*, *qnrS*, *dfrA1*, and *fosA*	[55–57]
	Tunisia	2018 *(mcr*-1 to *mcr*-8)	20	*mcr*-1 (15 *E*. *coli*)	ST57, ST398, ST117, and ST2973 (A, B1, B2, and D)	IncP, IncFIB, and IncI1	*Int*1, *bla*_CTX-M-1_, *bla*_TEM-1b_, *bla*_SHV-12_, *tet*(*A*), *cmlA*, *sul1*, *sul3*, *qacE∆1*, *dfrA1 aadA1*,	[34]
Raw milk/cheese	Egypt	2016-2017 (*mcr*-1)	4	*mcr*-1 (1 *E*. *coli*)	ST69 (*eilA*, *lpfA*, *gad*, *air*, *astA*, *iss*, *fimH*, *iutA*, *kpsMIII*, *kpsTIII*, and *iutA*)	IncHI2 and IncHI2A (IS*Apl1*)	*Int*1, *dfrA17*, *aadA1*, *aadA2*, *aadA5*, *aph*(*3*′)*-Ia*, *strA*-*strB*, *sul2*, *sul3*, *bla*_TEM-1_, *cmlA1*, *floR*, *qacH2*, and *tet*(*A*)	[54]
	Tunisia	2017-2018 (*mcr-1* to *mcr-5*)	5	*mcr*-1 (4 *E*. *coli*)	ST1642 and ST10 (B1)	IncP and IncFIB	*IntI*1, *bla*_CTX-M-1_, *bla*_TEM-1b_, *qacE∆1*-*sul1*, *tet*(A), *cmlA*, *sul3*, *dfrA1*, and *aadA1*	[50]

*mcr*: mobile colistin resistance gene; -: no data; additional resistance traits: resistance factors identified in one or pooled *mcr*-positive isolates; sequence type: Warwick multilocus sequence type of *mcr*-habouring *E*. *coli* isolates; virulence genes: genes detected in *E*. *coli* isolates; plasmid: plasmid types identified in one or pooled *mcr*-positive isolates; Inc.: incompatibility; IS: insertion sequence.

**Table 3:** Studies reporting plasmid-mediated colistin resistance in the environment in Africa.

Sources	Country	Year of isolation (*mcr* gene assayed)	Number of tested for *mcr*	Identified gene/variant (number of organism)	Sequence type (virulence genes)	Plasmid	Additional resistance traits	Reference
Wild monkey	Algeria	2016 (*mcr*-1)	1	*mcr*-1 (1 *E*. *coli*)	ST405 (*fyuA*)	-	*bla*_CTX-M-15_, *bla*_TEM-1_, and *qnrB19*	[67]
Wild/migratory birds	Egypt	2017-2018 (*mcr*-1 and *mcr*-2)		*mcr*-1 (9 *E*. *coli*, 3 *Klebsiella pneumoniae*, and 3 *Pseudomonas aeruginosa*) and *mcr*-2 (1 *P*. *aeruginosa*, 1 *E*. *coli*, and 1 K. *pneumoniae*)	-	-	-	[69]
Wastewater	South Africa	2018 (*mcr*-1)	65	*mcr*-1 (31 *E*. *coli*)	-	-	-	[65]
	Tunisia	2017-2019 (*mcr*-1 to *mcr*-8)	7	*mcr*-1 (1 *E*. *coli*)	ST8900 and A	IncFIB and IncP-1	*dfrA12*, *aadA2*, *qacEΔ1*, *sul1*, *tet*(*A*), *cmlA*, and *sul3*	[71]
Water (sea, river and irrigation)	Egypt	*mcr-1* and *mcr-2*		*mcr*-1 (2 *E*. *coli* and 1 *K*. *pneumoniae*) and *mcr*-2 (1 *E*. *coli* and 1 *K*. *pneumoniae*)	-	-	-	[69]
	Algeria	2016 (*mcr*-1 to *mcr*-5)	287	*mcr*-1 (3 *E*. *coli*)	ST23, ST115, and ST345	IncFII and IncI2A	*bla*_TEM-1B_, *aac*(*3*)*-IId*, *aadA1*, *aadA2*, *aph*(*30*)*-Ia*, *aph*(*3*)*-Ib*, *aph*(*6*)*-Id*, *mph*(*A*), *cml*, *sul1*, *sul3*, *tet*(*A*), *dfrA*, and *dfrA14*	[61, 68, 75]
Animal manure and soil	Algeria	2016-2018 (*mcr*-1 to *mcr*-5)	72	*mcr*-1 (4 *E*. *coli*) and *mcr*-3 (3 *E*. *coli*)	ST405, ST155, and ST10	-	-	[61]

*mcr*: mobile colistin resistance gene; -: no data; additional resistance traits: resistance factors identified in one or pooled *mcr*-positive isolates; sequence type: Warwick multilocus sequence type of all *mcr*-carrying *E*. *coli* isolates; virulence genes: genes detected in *E*. *coli* isolates; plasmid: plasmid types in one or pooled *mcr*-bearing isolates; Inc: incompatibility.

**Table 4:** Studies reporting plasmid-mediated resistance in isolates from humans in Africa.

Country	Year of isolation (*mcr* gene assayed)	Number of isolate tested for *mcr*	Identified gene/variant (number of organism)	Sequence type and/or phylogroup (virulence genes)	Plasmid (associated insertion sequence)	Additional resistance trait	References
South Africa	2013-2016 (*mcr*-1 and *mcr*-9)	68	*mcr*-1 (17 *E*. *coli* and 5 *Klebsiella*), *mcr*-9.1 (3 *Enterobacter hormaechei*)	ST10, ST57, ST624, ST101, ST226, and ST1007 (*pilIV*)	IncI2, IncHI2, and IncX4 (IS*ApI1* and IS*28*)	*Int*127, *Int*46, *Int*615, *bla*_TEM-1_, *bla*_CTX-M-55_, *bla*_CMY-2_*bla*_ACT-56_, *bla*_SHV-12_, *bla*_ACT-15_, *bla*_TEM-1B_, *bla*_TEM-1A_, *bla*_CTX-M-15_, *bla*_OXA-1_, *bla*_OXA-9_, *floR*, *aac(3)-II*, *aac*(*6*′)*-IIc*, *aac*(*6*′)*-Ib3*, *aac*(*6*′)*Ib-cr*, *aac*(*3*)*-II*, *aac*(*3*)*-IIa*, *aadA1*, *aadA2*, *aph*(*3*′)*-Ib*, *aph*(*3*′)*-Ia*, *aph*(*6*)*-Id*, *arr*, *catA*, *catA2*, *dfrA19*, *ere*(*A*), *fosA*, *∆qacE1*, *sul1*, *sul2*, *tet*(*D*), *aacA4*, *strA*, *aac*(*6*′)*-IIc*, *strB*, *qnrA1*, *dfrA12*, and *catB3*	[83–87]
Egypt	2015-2019 (*mcr*-1, *mcr*-2, and *mcr*-9)	432	*mcr*-1 (4 *E*. *coli*, 3 *Klebsiella pneumoniae*, 9 *Pseudomonas aeruginosa*, and 2 *Acinetobacter baumannii*) and *mcr*-9 (1 *E*. *hormaechei*)	ST1011, *Enterobacter*: ST133; *Klebsiella*: ST11	IncHI2 (IS*1* and IS*903*)	*Int*1, *bla*_TEM-1B_, *bla*_ACT-7_, *bla*_CTX-M-15_, *bla*_NDM-1_, *dfrA12-orf12-aadA2*, *bla*_VIM-4_, *fosA*, *sul1*, *qnrA1*, *dfrA1*, *aac*(*6*′)*-Il*, *aadA23*, *aadA2b*, *ant*(*2*^″^)*-Ia*, and *mgrB*	[69, 89–95, 100, 101]
Algeria	2011-2018 (*mcr*-1 to *mcr*-5 and *mcr*-8)	372	*mcr*-1 (11 *E*. *coli* and 1 K*lebsiella pneumoniae*) and *mcr*-8 (1 *K*. *pneumoniae*)	ST405, ST93, ST453, ST648, ST656, ST10, and ST155; *K*. *pneumoniae*: ST788; *Klebsiella*: ST336 (*ybtAEPQSTUX*, *irp1*, *irp2*, and *fyuA*)	IncFIB and IncP	*bla*_OXA-48_, *bla*_SHV-1_, *bla*_CTX-M-15_, *bla*_SHV-94_, *bl*a_OXA-1_, *bla*_TEM-_1, *bla*_TEM-1B_, *aph*(*3*′)*-la*, *aac*(*6*′)*-lb-cr*, *aph*(*3*′)*-lb*, *aph*(*6*)*-ld*, *oqxB*, *oqxA*, *fosA*, *catB3*, *sul2*, and *dfrA14*	[28, 60, 76, 77, 96, 97]
Morocco	2013-2014 (*mcr*-1)	2	*mcr*-1 (2 *E*. *coli*)	ST602 and ST11300		*bla*_TEM-1_	[60]
Congo	2017-2018 (*mcr*-1)	14	*mcr*-1 (3 *Pseudomonas aeruginosa*, 1 *P*. *luteola*, and 1 *P*. *putida*)	-	-	-	[66]
Tanzania	2017 (*mcr*-1 and *mcr*-2)	42	*mcr*-1.1 (11 *E*. *coli*)	ST46 (*cma*, *iroN*, and *iss*)	IncX4	*bla*_CTX-M-15-like_, *bla*_CTX-M-1-like_, *bla*_CTX-M-9-like,_*bla*_DHA,_*bla*_TEM-1B_, *aph*(*300*)*-Ib*, *aph*(*6*)*-Id*, *aadA5*, *mdf*(*A*), *mph*(*A*), *sul1*, *sul2*, *tet*(*A*), *dfrA17*, mutations in ParC, GyrA, ParE, and pmrB	[81]
Sudan	2016-2019 (*mcr*-1)	235	*mcr*-1 (7 *E*. *coli*, 1 *Pseudomonas*, and 5 K. *pneumoniae*)	-	-	-	[78, 79]
Kenya	2017 (*mcr*-1 to *mcr*-8)	1	*mcr*-8 (1 *K*. *pneumoniae*)	ST15	IncHI1B and IncR	*armA*, *aac*(*3*)*-IV*, *aac*(*6*′)*-Ib-cr*, *aadA1*, *aadA16*, *aadA2*, *aph*(*3*^″^)*-Ib*, *aph*(*3*′)*-Ia*, *aph*(*4*)*-Ia*, *aph*(*6*)*-Id*, *bla*_DHA-1_, *bla*_SHV-28_, *oqxA*, *oqxB*, *qnrB2*, *qnrB4*, *cmlA1*, *floR*, *ARR-3*, *dfrA27*, *fosA*, *mdf*(*A*), *mph*(*E*), *msrE*, *sul1*, and *sul3*	[80]
Nigeria	2016-2019 (*mcr*-1 to *mcr*-8)	130	*mcr*-1 (4 *E*. *coli* and 3 K. *pneumoniae*) and *mcr*-8 (3 *K*. *pneumoniae*)	-	IncX4	*QnrS1*, *oqxB*, *tet*(*A*), *tet*(*G*), *sul1*, *sul3*, *bla*_SHV-11_, *bla*_TEM-1_, and *bla*_SHV-1_	[40, 82]

*mcr*: mobile colistin resistance gene; -: no data; additional resistance traits: resistance factors identified in one or pooled *mcr*-positive isolates; virulence genes: genes detected in *E*. *coli* isolates; sequence type: Warwick multilocus sequence type of all *mcr*-carrying *E*. *coli* isolates; plasmid: plasmid types in one or pooled *mcr*-bearing isolates; Inc: incompatibility.
